# Influence of Online Sessions via a Deep Brain Stimulation Device: Prospective, Single-Arm, Longitudinal, Nonrandomized Self-Controlled Cohort Study

**DOI:** 10.2196/80223

**Published:** 2026-06-09

**Authors:** Ryoma Morigaki, Hideo Mure, Kouki Takasuka, Joji Fujikawa, Toshiro Shinmen, Shin Tarui, Masataka Hatanaka, Keisuke Shinohara, Taku Matsuda, Kazuhisa Miyake, Yasushi Takagi

**Affiliations:** 1 Department of Advanced Brain Research, Graduate School of Biomedical Sciences, Tokushima University, 3-18-15 Kuramoto-chou, Tokushima, 770-8503, Japan, 81 886337149 ext 3246, 81 886329464; 2 Parkinson’s Disease and Dystonia Research Center, Tokushima University Hospital, Tokushima, Japan; 3 Department of Neurosurgery, Graduate School of Biomedical Sciences, Tokushima University, Tokushima, Japan; 4 Center for Neuromodulation, Kurashiki Heisei Hospital, Kurashiki, Japan; 5 Department of Neurological Surgery, Kurashiki Heisei Hospital, Kurashiki, Japan

**Keywords:** Parkinson disease, telemedicine, deep brain stimulation, remote consultation, motor disorders, global burden of disease, caregiver burden

## Abstract

**Background:**

Deep brain stimulation (DBS) is widely performed in patients with advanced Parkinson disease (PD). Recent advances in technology have facilitated remote programming of DBS devices, reflecting an emerging trend in neuromodulation approaches, and offering a potential framework for patient-centered care. These online sessions for patients with PD who underwent de novo implantation of DBS devices have been reported to be safe and effective, similar to in-clinic sessions. Currently, evidence for patients with chronically implanted DBS devices remains limited.

**Objective:**

This study aimed to evaluate the safety, feasibility, and preliminary efficacy of online sessions for patients with PD who had chronically implanted DBS devices, and to determine whether this approach could reduce patient burden without compromising clinical management.

**Methods:**

A prospective, single-arm, longitudinal, nonrandomized, self-controlled cohort study was conducted at 2 centers in Japan. Eighteen patients with PD who had chronically implanted DBS devices were enrolled. Two in-clinic sessions were substituted with online sessions. The Movement Disorder Society Unified Parkinson’s Disease Rating Scale Part III scores were assessed at baseline, after in-clinic sessions, and online sessions. The Multidimensional Evaluation Scale for Patient Impression Change—Japanese version, Patient’s Global Impression of Change, and Clinical Global Impression of Change were each administered after both in‑clinic and online sessions. The Telehealth Usability Questionnaire and the Online Usability Questionnaire for Programming were evaluated once after online sessions.

**Results:**

The Movement Disorder Society Unified Parkinson’s Disease Rating Scale Part III scores did not differ significantly across the 3 assessments. The Multidimensional Evaluation Scale for Patient Impression Change—Japanese version scores revealed that no items but item 3, “overall sleep,” significantly worsened after online sessions. The Patient’s Global Impression of Change and Clinical Global Impression of Change scores were comparable between in-clinic and online sessions. The Telehealth Usability Questionnaire scores indicated that online sessions were generally favorable, although the "Reliability" was rated comparatively lower. The mean total time and cost saved per visit were 280 minutes and 7974 yen (US $1≈155 JPY), respectively. One patient experienced a fall and lumbar compression fracture during the online visit period and dropped out of the study because of hospitalization.

**Conclusions:**

Online sessions may be a feasible option for a subset of patients with PD carrying chronically implanted DBS devices. Although online sessions cannot fully replace in-person assessments, they may serve as a practical alternative when adequate caregiver support, sufficient digital literacy, and a reliable connection are ensured. Clinicians should note that sleep-related symptoms may worsen in some patients. While time and cost savings are not direct indicators of care quality, these benefits still provide meaningful support for patients and caregivers. Overall, online sessions may complement routine follow-up in stable patients undergoing chronic DBS therapy.

## Introduction

### Online Sessions as an Emerging Modality in Parkinson Disease Care

Parkinson disease (PD) is a multisystem neurodegenerative disorder characterized by motor and nonmotor symptoms [1]. Due to its progressive nature, the burden on patients and their caregivers increases over time. Recent studies have demonstrated the effectiveness of online sessions including remotely supervised exercise and telerehabilitation for patients with PD [2-6]. The advancement and prevalence of remote technologies, in part owing to the COVID-19 pandemic, have promoted remote programming and surveillance in patients who have undergone deep brain stimulation (DBS) [7-18]. Several studies have shown that online sessions are highly effective, particularly in reducing the cost and time burden for patients with PD who underwent DBS, as well as their caregivers [8-11,15-18].

### Current Evidence on Online Sessions for DBS

In patients with de novo DBS, 2 randomized studies and several observational studies have shown that remote programming is safe, effective, and comparable with in-clinic adjustments, with high satisfaction rates [7,11,12,15,16,18]. These findings suggest that online sessions can support early postoperative management and reduce the need for frequent hospital visits. In contrast, evidence in patients who had chronically implanted DBS devices is more limited. Retrospective and cross-sectional studies indicate that remote programming may reduce travel distance, time, and financial burden, and that patient satisfaction is generally favorable [8,10,13,14,17]. However, these studies are heterogeneous, mostly observational, and lack prospective evaluation.

### Rationale and Objective for This Study

The clinical needs of patients who had undergone chronic DBS differ from those of de novo cases, as long-term follow-up typically involves clinical review, medication management, and device checks rather than frequent parameter adjustments. Thus, despite accumulating evidence, prospective, population-specific data on the safety and feasibility of online sessions in chronic DBS care remain insufficient. To date, no prospective study has evaluated online sessions in patients with PD who underwent chronic implantation of DBS devices, and detailed data are still lacking. Given these gaps, we aimed to explore whether online sessions could be implemented safely and feasibly in patients with chronic DBS devices, while assessing their preliminary efficacy in maintaining clinical stability and reducing patient burden—particularly travel-related demands—without compromising routine clinical management.

## Methods

### Study Design

**Figure 1.**
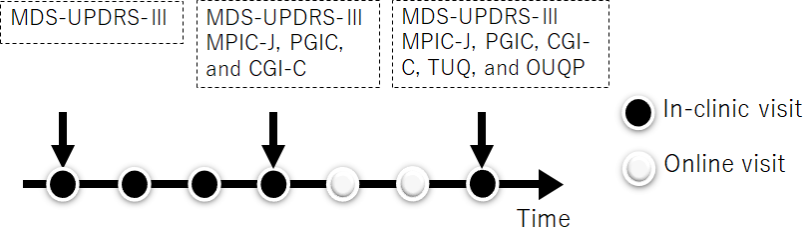
Schematic figure explaining the planning of the experiment. CGI-C: Clinical Global Impression of Change; MDS-UPDRS-III: Movement Disorder Society Unified Parkinson’s Disease Rating Scale Part III; MPIC-J: Multidimensional Evaluation Scale for Patient Impression Change—Japanese Version; OUQP: Online Usability Questionnaire for Programming; PGIC: Patient’s Global Impression of Change; TUQ: Telehealth Usability Questionnaire.

A prospective, single-arm, longitudinal, multicenter, nonblinded, nonrandomized, and self-controlled cohort trial was conducted at Tokushima University Hospital and Kurashiki Heisei Hospital in Japan between 2023 and 2024. All participants had been treated with in-clinic sessions, followed by 2 consecutive online sessions (Figure 1).

### Ethical Considerations

This study was approved by the Ethics Committee of Tokushima University (approval number 4409, date of approval August 28, 2023) and the Ethics Committee of Kurashiki Heisei Hospital (approval number R05-008, date of approval July 4, 2023) in accordance with the Declaration of Helsinki. The participants received detailed study information and signed informed consent forms before enrollment. To protect participant privacy, all data were deidentified, stored on secure institutional servers, and accessible only to authorized study personnel. No audio or video recordings were collected during online sessions, and all telehealth communications were conducted through encrypted, institution‑approved platforms. Participants did not receive financial compensation for study participation.

### Participants

All participants met the following inclusion criteria: (1) adult patients (aged 18 years and older) diagnosed with PD, (2) those who had received bilateral subthalamic nucleus (STN) DBS using the Infinity DBS system with the NeuroSphere Virtual Clinic remote care (Abbott), (3) those who had a follow-up period of at least 3 years postsurgery, (4) those who had never experienced online sessions, and (5) those who are able to operate the online system with or without caregivers and consented to be included in this study. For the sample size calculation, we estimated that 15 participants would provide 80% power (with a 5% probability of type I error) and a large effect size using G*Power 3.1. Therefore, we set the target sample size to at least 17 participants and estimated a dropout rate of 10%.

### Assessments

#### Primary Outcomes

##### Clinical Motor Assessments

The Movement Disorder Society Unified Parkinson’s Disease Rating Scale Part III (MDS-UPDRS-III) scores were recorded before and after each of the 2 in-clinic sessions and 2 online sessions, using identical in-person, face-to-face assessment protocols conducted by the same evaluators with active DBS stimulation and on medications (Figure 1).

##### Safety Assessments

Safety was evaluated throughout the study period by monitoring adverse events. Participants were reported any new or worsening medical conditions during both in-clinic and online sessions. All adverse events, including falls, injuries, or any clinical deterioration, were recorded by the investigators, regardless of their perceived relationship to the online sessions.

### Secondary Outcomes

#### Patient-Reported Assessments

Three self-reported numerical rating scales, including the Multidimensional Evaluation Scale for Patient Impression Change—Japanese version (MPIC-J) [19,20], Patient’s Global Impression of Change (PGIC) [21], and Clinical Global Impression of Change (CGI-C) [22,23], were obtained before and after the 2 online sessions (Figure 1). Two self-reported numerical rating scales, the Telehealth Usability Questionnaire (TUQ) [24] and Online Usability Questionnaire for Programming (OUQP), were evaluated after the 2 online sessions (Figure 1).

The MPIC assesses 8 domains (overall status, pain, sleep, mood, physical functioning, coping with pain, managing pain flare-ups, and medication effectiveness) with 7 ratings: 1 = very much improved, 2 = much improved, 3 = minimally improved, 4 = no change, 5 = minimally worse, 6 = much worse, and 7 = very much worse [19]. PGIC was rated on the 7-point scale: 1 = no change (or condition has got worse); 2 = almost the same, hardly any change at all; 3 = a little better but no noticeable change; 4 = somewhat better, but the change has not made any real difference; 5 = moderately better, and a slight but noticeable change; 6 = better, and a definite improvement that has made a real and worthwhile difference; and 7 = a great deal better, and a considerable improvement that has made all the difference [25]. The CGI-C was also rated on the 7-point scale: 1 = very much improved, 2 = much improved, 3 = minimally improved, 4 = no change from baseline (the initiation of treatment), 5 = minimally worse, 6 = much worse, and 7 = very much worse since the initiation of treatment [22,23]. The TUQ has 6 subdomains, with each consisting of 3‐4 questionnaires with 5 answers: “Strongly disagree,” “Disagree,” “Neutral,” “Agree,” and “Strongly Agree.” Subdomains are “Usefulness,” “Ease of use and learnability,” “Interface quality,” “Interaction quality,” “Reliability,” and “Satisfaction and future use” [24]. The OUQP was developed by modifying the assessment items originally proposed by Deer et al [9], a 6-item questionnaire with cost and time savings. Patient satisfaction was assessed using 3 criteria: answering yes/no or neither. Patients were additionally invited to comment on any challenges or difficulties they experienced during the online sessions.

#### Clinician-Reported Assessments

Clinician satisfaction with the online sessions was assessed using 5 criteria in the OUQP. Clinicians also provided qualitative feedback regarding any operational or clinical difficulties encountered during remote programming.

#### Cost and Time Burden Evaluation

Patients were also asked to report the cost and time burdens associated with their in-clinic sessions. The OUQP included items assessing perceived time and cost savings during online sessions. Patient satisfaction with online sessions was assessed using 3 yes/no/neither questions, and patients were asked to describe any difficulties encountered. The Japanese version of PGIC, CGI-C, TUQ, and OUQP is provided in Multimedia Appendix 1.

### Statistics

All measured data are presented as mean (SD). The 2-tailed *t* test (matched pairs) was used to analyze the 2 groups when the normality of distribution was verified by the Shapiro-Wilk test. If normality was not confirmed, the Wilcoxon rank-sum test was used. Significance and effect size were determined using Hedges *g* when normality was observed, or Cliff δ when it was not. The Friedman test was applied to compare repeated measures of MDS-UPDRS-III across the 3 assessment points and to evaluate seasonal variation in exposure days across 4 seasons (spring, summer, autumn, and winter).

For any MPIC-J items that showed statistically significant pre-post differences, additional exploratory analyses were conducted to evaluate the clinical relevance of the observed changes. Specifically, the minimally important change (MIC) was estimated using the MPIC-J 7-point global change scale as an anchor; receiver operating characteristic (ROC) analysis was performed to identify the optimal cutoff discriminating subjective worsening, and distribution-based estimates (0.5 SD) were also calculated. Correlation analyses were performed to explore associations between changes in clinical outcomes and candidate explanatory variables. Point-biserial correlation coefficients (*r*) were used for sex, which was treated as a dichotomous variable. For all other continuous or ordinal variables, Spearman rank correlation coefficients (*ρ*) were calculated. All statistical analyses were performed using R (version 4.2.1; R Core Team) [26], and the significance level was set at *P*<.05.

## Results

### Overview of Participants

**Figure 2.**
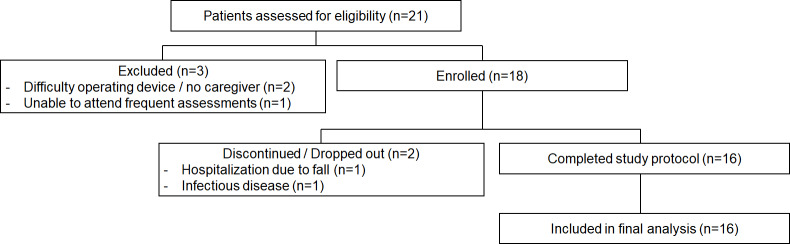
Participant flow diagram. A total of 21 patients were screened; 3 were excluded due to difficulty operating the device or inability to attend frequent assessments. Eighteen patients were enrolled, of whom 2 were discontinued because of hospitalization or infectious disease. Sixteen patients completed all assessments and were included in the final analysis.

Between August 2023 and April 2024, 21 patients were screened for eligibility from 2 hospitals. Three patients were excluded from the study for the following reasons: 2 patients had difficulty operating the equipment and no familial support, and 1 patient found it challenging to attend frequent assessment appointments. Eighteen patients were enrolled, of whom 2 discontinued participation because of hospitalization and infectious disease. The dropout rate was 11% (2/18). All data points were complete, and no missing values were identified throughout the dataset (Figure 2).

The demographic profile was as follows: 50% (8/16) were female, average age was 67.1 (SD 8.4) years (95% CI 62.6‐71.5), average Hoehn-Yahr Scale score was 3.7 (SD 1.0; 95% CI 3.1‐4.2), average duration of illness was 15.8 (SD 4.4) years (95% CI 13.4‐18.1), average duration after bilateral STN-DBS was 6.6 (SD 1.9) years (95% CI 5.5‐7.6), and the average distance from the resident to the clinic was 42.8 (SD 49.7) kilometers (95% CI 16.3‐69.3; Table 1). There was no difference in the duration between each assessment visit of in-clinic and online sessions (mean 138.4, SD 64.5 days, 95% CI 104.1‐172.8 vs mean 152.6, SD 57.2 days, 95% CI 122.2‐209.8; *P*=.53; Hedges *g*=0.220). Friedman tests revealed no significant seasonal variation in exposure days across the 4 seasons for in-clinic sessions (mean 56.8, SD 33.2 days, 95% CI 39.0‐74.6; *χ*²_3_=6.73; *P*=.081; Kendall *W*=0.140) or online sessions (mean 54.7, SD 36.2 days, 95% CI 35.8‐73.6; *χ*²_3_=6.83; *P*=.078; Kendall *W*=0.143). No adjustments were made to antiparkinsonian medications or to any sleep-affecting drugs throughout all study periods.

**Table 1.** Characteristics of 16 participants.

Number	Sex	Age (years; mean 67.1, SD 8.4 years)	Hoehn-Yahr grade (mean 3.7, SD 1.0)	Disease duration (years; mean 15.8, SD 4.4 years)	Duration after surgery (years; mean 6.6, SD 1.9 years)	Distance from the residence (km; mean 42.8, SD 49.7 km)	Travel time to the clinic (minutes; mean 148.4, SD 81.1 minutes)	Travel expenses for medical visits (JPY^a^; US $1≈155 JPY; mean JPY 4365.0, SD JPY 7472.9)	Caregivers (mean 0.9, SD 0.6), n
1	F^b^	67	3	14	5	39.9	120	2000	1
2	M^c^	56	2	18	10	48.1	120	3000	0
3	F	76	5	11	5	71.6	240	10,000	1
4	F	65	4	9	5	15.1	80	300	1
5	F	72	3	10	5.5	62.5	180	1000	0
6	F	78	5	19	6.5	38.7	120	30,000	1
7	M	75	4	21	4.5	3.0	25	3000	1
8	F	50	2	25	8	13.5	60	390	0
9	F	66	4	18	5	25.6	50	600	1
10	M	68	3	12	5.5	135.0	240	10,000	0
11	M	57	3	12	5.5	24.2	150	750	1
12	M	65	3	13	5.5	17.0	180	2000	1
13	M	60	5	17	10	178.0	360	2000	1
14	M	66	5	17	9	2.0	150	2000	2
15	F	80	4	17	6	1.8	180	300	2
16	M	72	4	19	9	8.6	120	2500	1

a JPY: Japanese yen.

b F: female.

c M: male.

### Primary Outcome

#### Motor Assessments

All 16 patients completed all the assessments. Motor symptoms were assessed by using MDS-UPDRS-III. The average total scores were 24.9 (SD 21.1; 95% CI 13.7‐36.2), 28.6 (SD 22.5; 95% CI 16.6‐40.6), and 28.8 (SD 22.0; 95% CI 17.1‐40.6) at baseline, after in-clinic session, and after online session. Friedman test revealed no differences between the groups (*P*=.27, Friedman *χ*²_2_=2.63). Comparison of the rate of change (%) in total MDS-UPDRS-III scores between in-clinic and online sessions revealed n significant difference (mean −19.3%, SD 33.4% vs mean −3.2%, SD 35.3%; *P*=.31; Hedges *g*=0.474). These findings indicate no statistically or clinically meaningful worsening of motor symptoms in online sessions compared with in-clinic sessions.

#### Safety

One patient experienced a fall and lumbar compression fracture during the online visits period. and dropped out from the study because of hospitalization. No other adverse events were observed.

### Secondary Outcomes

#### Patient-Reported Assessments

In comparison of MPIC-J scores of in-clinic and online sessions, no items but MPIC-J item 3 (overall sleep) showed significant difference (mean 3.31, SD 0.85 vs mean 4.19, SD 0.88; *P*=.013, Cliff δ=−0.49) (Table 2). To further examine the clinical relevance of this change, exploratory analyses were performed for MPIC-J item 3. The mean change of 0.88 points exceeded the distribution-based threshold of 0.5 SD (=0.43), indicating a moderate magnitude of change. Using the MPIC-J 7-point global change scale as an anchor, participants who reported “minimally worse” sleep (MPIC-J score=5; n=4) showed a mean increase of 2.0 points in MPIC-J item 3, representing a reference value for the MIC in the worsening direction. ROC analysis, in which MPIC-J scores were dichotomized into “worsened” (5-7) and “not worsened” (1-4), identified a 1-point increase in MPIC-J item 3 as the optimal cutoff for discriminating subjective worsening (sensitivity 1.00 and specificity 0.73). Taken together, although the observed mean change exceeded the distribution-based threshold, it did not reach the ROC-derived cutoff (1 point) or the anchor-based MIC estimate (2 points), suggesting that the magnitude of sleep deterioration may be limited in its clinical significance.

Correlation analyses were conducted to evaluate whether the change in MPIC-J item 3 was associated with baseline characteristics, exposure-related factors such as the duration of in-person and online sessions, or changes in other clinical measures (Tables S1-S3 in Multimedia Appendix 2). No baseline characteristics showed significant correlations with the change in MPIC-J item 3. The duration of in-person sessions was not associated with ΔMPIC-J item 3 (*ρ*=0.0077, *P*=.98). In contrast, the duration of online sessions showed a strong positive correlation with ΔMPIC-J item 3 (*ρ*=0.71, *P*=.0021), and this association remained significant after Bonferroni correction. Regarding exposure-related and change-based variables, ΔMDS-UPDRS-III during the in-person period showed a positive correlation with ΔMPIC-J item 3 (*ρ*=0.52, *P*=.038), and ΔMPIC-J item 7 (coping with pain) was also correlated with ΔMPIC-J item 3 (*ρ*=0.54, *P*=.031). ΔMPIC-J item 2 (pain) showed a similar trend (*ρ*=0.44, *P*=.086). This implies that patients with greater clinical instability were more likely to experience sleep deterioration when they spent a longer proportion of the follow-up period in online sessions. However, none of these correlations remained significant after Bonferroni correction, indicating that these associations should be interpreted as exploratory and may reflect covarying deterioration across multiple domains rather than robust predictors of sleep worsening.

There was no difference between in-clinic and online sessions for both PGIC and CGI-C (mean 1.88, SD 1.05, 95% CI 1.31‐2.44 vs mean 1.81, SD 0.95, 95% CI 1.31‐2.32, *P*=.73, Cliff δ=0.01 for PGIC-1; mean 5.19, SD 0.81, 95% CI 4.76‐5.62 vs mean 5.06, SD 1.25, 95% CI 4.40‐5.73, *P*≥.99, Cliff δ=−0.10 for PGIC-2; and mean 3.81, SD 0.73, 95% CI 3.43‐4.20 vs mean 3.63, SD 0.70, 95% CI 3.25‐4.00, *P*=.53, Cliff δ=0.15 for CGI-C).

**Table 2.** MPIC-J^a^ scores on in-clinic and online sessions.

Items	In-clinic			Online			*P* value	Cliff δ
	Mean (SD)		95% CI	Mean (SD)		95% CI		
Overall status	3.94 (0.97)		3.42‐4.45	4.31 (0.98)		3.79‐4.85	.27	−0.22
Overall pain	3.94 (0.66)		3.59‐4.29	4.25 (1.03)		3.70‐4.80	.13	−0.13
Overall sleep	3.31 (0.85)		2.86‐3.76	4.19 (0.88)		3.72‐4.66	.01^b^	−0.49
Overall mood	3.94 (1.30)		3.25‐4.63	4.19 (0.88)		3.72‐4.66	.45	−0.14
Overall physical functioning	4.19 (1.38)		3.45‐4.92	4.19 (0.88)		3.72‐4.66	.≥.99	0.02
Overall ability to cope with my pain	3.94 (0.90)		3.46‐4.42	4.00 (0.71)		3.62‐4.38	.65	−0.05
Overall ability to manage pain flare-ups	3.63 (0.70)		3.25‐4.00	3.88 (0.86)		3.42‐4.33	.16	−0.20
Overall effectiveness of my medication	3.75 (0.97)		3.23‐4.27	4.19 (0.95)		3.68‐4.69	.10	−0.18

a MPIC-J: Multidimensional Evaluation Scale for Patient Impression Change—Japanese version.

b *P*<.05.

#### Usability

The TUQ revealed that more than 70% of the patients responded with “Neutral,” “Agree,” or “Strongly agree” to all items except item 15, “I think the visits provided over the telehealth system are the same as in-person visits.” For this item, 25% (4/16) of patients selected “Disagree,” and 6.25% (1/16) of patients selected “Strongly disagree” (Table 3). There were 6 subdomains in the TUQ: “usefulness” (items 1‐3), “ease of use and learnability” (items 4‐6), “interface quality” (items 7‐10), “interaction quality” (items 11‐14), “reliability” (items 15‐17), and “satisfaction and future use” (items 18‐21). In favor of online sessions, selecting “Strongly agree” or “Agree” was 70.8% (34/48) in “usefulness” domain, 85.4% (41/48) in “ease of use and learnability” domain, 53.1% (34/64) in “interface quality” domain, 62.5% (40/64) in “interaction quality” domain, 37.5% (18/48) in “reliability” domain, and 62.5% (40/64) in “satisfaction and future use” domain (Tables3  4). Overall, patients found the online sessions useful, feasible, and satisfactory, although some concerns about reliability were noted.

**Table 3.** Telehealth Usability Questionnaire.

Number	Items	Strongly disagree	Disagree	Neutral	Agree	Strongly agree
Usefulness, n (%)						
1	Telehealth improves my access to health care services	0 (0.0)	3 (18.8)	4 (25.0)	3 (18.8)	6 (37.5)
2	Telehealth saves me time traveling to a hospital or specialist clinic	0 (0.0)	1 (6.25)	0 (0.0)	3 (18.8)	12 (75.0)
3	Telehealth provides for my health care needs	1 (6.25)	0 (0.0)	5 (31.3)	6 (37.5)	4 (25.0)
Ease of use and learnability, n (%)						
4	It was simple to use this system	0 (0.0)	2 (12.5)	0 (0)	7 (43.8)	7 (43.8)
5	It was easy to learn to use the system	0 (0.0)	1 (6.25)	2 (12.5)	6 (37.5)	7 (43.8)
6	I believe I could become productive quickly using this system	0 (0.0)	0 (0.0)	2 (12.5)	7 (43.8)	7 (43.8)
Interface quality, n (%)						
7	The way I interact with this system is pleasant	0 (0.0)	0 (0.0)	8 (50.0)	3 (18.8)	5 (31.3)
8	I like using the system	2 (12.5)	2 (12.5)	3 (18.8)	4 (25.0)	5 (31.3)
9	The system is simple and easy to understand	1 (6.3)	0 (0.0)	5 (31.3)	4 (25.0)	6 (37.5)
10	This system is able to do everything I would want it to be able to do	0 (0.0)	4 (25.0)	5 (31.3)	3 (18.8)	4 (25.0)
Interaction quality, n (%)						
11	I could easily talk to the clinician using the telehealth system	0 (0.0)	0 (0.0)	5 (31.3)	3 (18.8)	8 (50.0)
12	I could hear the clinician clearly using the telehealth system	1 (6.3)	0 (0.0)	2 (12.5)	6 (37.5)	7 (43.8)
13	I felt I was able to express myself effectively	0 (0.0)	1 (6.25)	7 (43.8)	4 (25.0)	4 (25.0)
14	Using the telehealth system, I could see the clinician as well as if we met in person	1 (6.3)	2 (12.5)	5 (31.3)	3 (18.8)	5 (31.3)
Reliability, n (%)						
15	I think the visits provided over the telehealth system are the same as in-person visits	1 (6.3)	4 (25.0)	3 (18.8)	3 (18.8)	5 (31.3)
16	Whenever I made a mistake using the system, I could recover easily and quickly	2 (12.5)	0 (0.0)	9 (56.3)	1 (6.3)	4 (25.0)
17	The system gave error messages that clearly told me how to fix problems	0 (0.0)	0 (0.0)	11 (68.8)	1 (6.3)	4 (25.0)
Satisfaction and future use, n (%)						
18	I feel comfortable communicating with the clinician using the telehealth system	0 (0.0)	4 (25.0)	4 (25.0)	3 (18.8)	5 (31.3)
19	Telehealth is an acceptable way to receive health care services	0 (0.0)	0 (0.0)	5 (31.3)	4 (25.0)	7 (43.8)
20	I would use telehealth services again	0 (0.0)	2 (12.5)	4 (25.0)	3 (18.8)	7 (43.8)
21	Overall, I am satisfied with this telehealth system	0 (0.0)	0 (0.0)	5 (31.3)	4 (25.0)	7 (43.8)

**Table 4.** Subdomains in Telehealth Usability Questionnaire.

Subdomains	In favor of in-clinic sessions	Neutral	In favor of online sessions
Usefulness, %	10.4	18.8	70.8
Ease of Use and Learnability, %	6.3	8.3	85.4
Interface Quality, %	14.1	32.8	53.1
Interaction Quality, %	7.8	29.7	62.5
Reliability, %	14.6	47.9	37.5
Satisfaction and Future Use, %	9.4	28.1	62.5

The OUQP revealed that 2 clinicians experienced no difficulty in using online devices and felt a reduced necessity for in-clinic sessions in 62.5% (10/16) of the patients; however, sometimes it was not easy to provide rapid treatment resolution (5/16, 31.3%; Table 5). The reasons were, in part, the inability of clinicians to touch patients directly (eg, inability to check the rigidity of extremities) and the anxiety of the patients for not consulting in person. Approximately 70% of patients with PD were satisfied with online sessions; however, the preference for online sessions was 37.5% (6/16), which was the same percentage as the preference for an in-clinic session.

**Table 5.** Online Usability Questionnaire for Programming.

Items	Yes	No	Neither
For physician, %			
Ability to establish an audiovisual connection	100.0	0.0	0.0
Achieving the clinical goals of a programming session	100.0	0.0	0.0
Ability to complete a session	100.0	0.0	0.0
Reduction in the need for an in-person follow-up after a remote session	62.5	18.8	18.8
Providing rapid treatment resolution	68.8	0.0	31.3
For patient, %			
Satisfied with a remote session	68.8	12.5	18.8
Preference for a remote session	37.5	37.5	25.0
Providing rapid treatment resolution	56.2	37.5	6.3

#### Reduction in Time and Cost Burdens

The mean time and cost per patient visit were 148.4 (SD 81.1 minutes; 95% CI 105.2‐191.6) and 4365.0 (SD 7472.9 Japanese yen [US $1≈155 JPY]; 95% CI 383.0‐8,347.0), respectively. Seventy-five percent of patients with PD required caregivers for their visit, irrespective of in-clinic or online sessions. The average number of caregivers attending the patients was 0.88 (SD 0.60; 95% CI 0.56‐1.19) per visit (Table 1). The average total time and cost saved per visit for patients and caregivers were calculated as 280.0 (SD 181.5 minutes; 95% CI 183.3‐376.7) and 7974.4 (SD 14,235.9 JPY [US $1≈155 JPY]; 95% CI 388.6‐15,560.1), respectively (Table 1).

## Discussion

### Main Findings in Patients Under Chronic DBS

Our study demonstrated the feasibility of online sessions using the NeuroSphere Virtual Clinic remote care system compared with the conventional in-clinic sessions. Motor symptoms did not change in either group, as assessed using the MDS-UPDRS-III. Motor stability in patients under chronic DBS is expected over short intervals, regardless of visit modality; therefore, the absence of deterioration might not be interpreted as evidence of clinical effectiveness. This study provides a prospective evaluation of the effect of online sessions on patients who have already been implanted with and under chronic DBS. To our knowledge, this is the first study to investigate the effects of online sessions using DBS devices in Japanese patients. While the prospective design improves data quality compared with retrospective reports, the single-arm nature of this study precludes definitive causal inferences.

The only adverse event occurred between the last in-clinic visit and the first online session, making it unlikely that the event was related to the online sessions. Overall, online sessions appeared to be feasible and clinically acceptable from a safety perspective for patients with PD who have chronically implanted DBS devices. Although the fall was not clearly attributable to online sessions, the inability to directly assess rigidity, balance, and gait remains an inherent limitation of remote care. As expected, there was a substantial reduction in both cost and time burden for patients and caregivers [8,9,11,15-18].

### Patient-Reported Outcomes and Usability

In this study, approximately half of the patients thought that online sessions were equivalent to in-clinic sessions, as reflected in TUQ item 15 and the OUQP. Around 70% of patients reported satisfaction with the online sessions (TUQ item 21 and OUQP; Tables3  5). Given that TUQ subdomain “Usefulness” refers to “the positive effects on clinical outcomes” or “reduction in clinical cost” [24], our results indicated that 70.8% (34/48) of the patients felt that online sessions had positive effects on clinical outcomes. Previous retrospective studies assessing patients with chronic DBS implantation indicated that 62% of patients preferred online visits [17]. A cross-sectional survey of teleprogramming after each online session for patients with chronic pain equipped with the NeuroSphere Virtual Clinic remote care showed that 93.8% (15/16) of the patients prefer the online session [9]. Similarly, a cross-sectional survey of teleprogramming following online sessions in patients with PD who had undergone de novo DBS implantation found that 100% of patients were satisfied with the online sessions and expressed a willingness to participate in future [11]. PD is progressive, and the burden of in-clinic visits for both patients and caregivers increases as symptoms worsen. In this context, online systems may help reduce that burden without negatively affecting patients’ core motor symptoms, although this requires confirmation in controlled studies.

### Sleep Deterioration and Its Clinical Interpretation

One unexpected finding was a potential signal of deterioration in sleep status in patients who received online sessions, which has not been previously reported (Table 2), although the observed change in sleep status consistently fell below the established MIC thresholds, indicating limited clinical relevance. Exploratory correlations suggested that patients with preexisting clinical instability—such as motor worsening or fluctuations in pain-related domains—were more likely to show sleep deterioration.

Interestingly, the duration of online sessions showed a strong association with ΔMPIC-J item 3. Because the online period varied among patients, this association is unlikely to reflect intentional differences in exposure to online sessions. Instead, it may imply that patients with greater instability were more susceptible to sleep deterioration when they spent a longer proportion of the follow-up period in online sessions. These exploratory correlations could imply a potential interaction between patient instability and the extent of online follow-up; however, these findings should be interpreted with caution. Taken together, these findings suggest a need for further investigation into whether sleep deterioration during online-based DBS management may not be solely attributable to the online modality itself but rather an emergence in clinically unstable patients who may instead benefit from shorter online follow-up and earlier return to in-person assessment.

### Comparison With Previous Studies and Added Value of This Study

According to a randomized controlled trial, online programming sessions for de novo implanted DBS systems resulted in approximately 38% shorter treatment duration compared with in-clinic optimization and increased patient access to therapy adjustments by nearly 1.9 times [18]. Given the strong therapeutic impact of the initial stimulation, there is concern that the added value of online sessions may be overlooked or inadequately assessed. In addition, Chen et al [15] reported the possibility that physicians may adjust medications less frequently during remote programming than during in-hospital programming for de novo DBS-implanted patients with PD. This raises the possibility of bias due to differences in dopamine levels. In our study, we targeted patients with PD with an average duration of 6.6 (SD 1.9, range 4.5‐10, median 5.5) years, representing the longest follow-up period reported in the literature. In contrast to the evidence in patients who have undergone de novo DBS, studies involving chronically implanted DBS systems have been retrospective or cross-sectional (Table 6). These reports suggest that remote programming can substantially reduce travel time and financial burden and is generally well accepted by patients [10,17] but lacked prospective evaluation of safety, usability, or clinical stability in long-term DBS management. Our study partially addresses this gap by providing preliminary prospective evaluation in patients who had chronically implanted DBS devices, offering a baseline for future comparative trials. It also offers the first population-specific data from Japan, using a device platform not previously examined in this context. By focusing on long-term DBS users, we examined the specific population for whom online sessions are presently feasible in real-world Japanese clinical settings. The inclusion of detailed usability measures (TUQ and OUQP) and the observation of a potential signal in sleep-related symptoms further add domain-specific insights not highlighted in earlier reports. Together, these findings suggest the feasibility and acceptable short-term safety profile of integrating online sessions into DBS care. Although time and cost savings are not direct care quality measures, our descriptive estimates—based on a typical frequency of 6 clinic visits per year—suggested that patients and caregivers might save approximately 28 hours and 47,844 JPY (US $1≈155 JPY).

**Table 6.** Summary of key studies on teleprogramming in deep brain stimulation for Parkinson disease.

Study (year)	Population type	Study design	Values, n^a^	Main outcomes	Key findings
PD^b^ with chronic DBS^c^					
Present study (2026)	PD with chronic STN-DBS^d^ (mean 6.6 years postoperatively)	Prospective, single-arm, multicenter, and self-controlled cohort	16	MDS-UPDRS-III^e^, MPIC-J^f^, PGIC^g^, CGI-C^h^, TUQ^i^, OUQP^j^, and time/cost savings	No motor worsening. Modest sleep deterioration. Saved 280 minutes and 7974 JPY^k^ (US $1≈155 JPY) per visit. 70% satisfaction.
Ma et al (2021) [10]	PD with STN-DBS (mean 27.0 months postoperatively)	Retrospective, single-center database review	90	Motor symptoms (UPDRS-III) and time/cost savings	Saved >12 hours (for 90% of patients) and >2000 CNY^l^ (US $1≈155 JPY) (for 76.7%) per visit. Feasible for long-term management.
Wan et al (2024) [17]	Patients with chronic DBS	Single-center cross-sectional survey	132	Patient burden, attitudes, and satisfaction	Teleprogramming reduces burden; 62% of chronic patients preferred online visits.
Pintér et al (2022) [14]	Movement disorders (PD, dystonia, and ET) with DBS (mean 5.8 years postoperatively)	Retrospective, single-center registry-based modeling	319	Modeled travel distance, time, cost, and caregiver burden	Saved 415.2 kilometers and 342.1 minutes per visit. Estimated to save 17 days of caregiver leave over 10 years.
Zhang et al (2021) [13]	Movement disorders (PD and dystonia) with DBS (median 6.7 months postoperatively)	Retrospective, national multicenter observational study	196	Frequency of requests, safety, and satisfaction	89% of 909 sessions were satisfactory. Essential for care during pandemic without serious adverse events.
Zhang et al (2020) [8]	Movement disorders (PD, dystonia, and ET^m^) with DBS (median 6.7 months)	Retrospective, single-center observational study	589	Implementation of Bluetooth technology	Safe and efficient parameter adjustments. Average travel distance saved: 1141 kilometers.
PD with de novo DBS					
Gharabaghi et al (2025) [18]	PD with de novo STN-DBS implantation	Prospective, multicenter, randomized controlled trial	38	Time to symptom improvement (PGI-C), QoL^n^, and safety	Remote group achieved clinical benefit 15.1 days faster than in-clinic group. Safe postoperative optimization.
Wan et al (2024) [16]	PD with de novo STN-DBS	Retrospective, single-center controlled study	18	ΔUPDRS-III, ΔPDQ-8^o^, and ΔLEDD^p^ (1-year follow-up)	No significant difference in motor improvement or LEDD reduction between in-clinic and online groups.
Chen et al (2022) [15]	PD with de novo STN-DBS	Retrospective, single-center controlled study	58	ΔMDS-UPDRS-III, LEDD reduction, and sessions	Improvement in motor scores was similar across remote, hospital, and combined groups.
Xu et al (2021) [12]	PD with de novo DBS	Retrospective, single-center observational study	28	Motor symptoms (UPDRS-III) and satisfaction	UPDRS-III scores significantly improved. 93.75% were satisfied with the procedure convenience.
Li et al (2017) [7]	PD with de novo bilateral STN-DBS	Prospective, single-center, randomized controlled trial	31	Safety and efficacy of remote programming	Remote adjustment was found effective compared with sham stimulation. Early safety verification.
Xu et al (2021) [11]	PD with de novo DBS	Prospective, single-center pilot study	18	Remote video-based outcome measures	Smartphone-based remote motor assessment (video) is a feasible pilot approach for post-DBS care.

a Number of patients who underwent remote programming and successfully completed the study assessments.

b PD: Parkinson disease.

c DBS: deep brain stimulation.

d STN-DBS: subthalamic nucleus–deep brain stimulation.

e MDS-UPDRS-III: Movement Disorder Society Unified Parkinson’s Disease Rating Scale Part Ⅲ.

f MPIC-J: Multidimensional Evaluation Scale for Patient Impression Change—Japanese version.

g PGIC: Patient’s Global Impression of Change.

h CGI-C: Clinical Global Impression of Change.

i TUQ: Telehealth Usability Questionnaire.

j OUQP: Online Usability Questionnaire for Programming.

k JPY: Japanese yen.

l CNY: Chinese yuan.

m ET: essential tremor.

n QoL: quality of life.

o PDQ-8: Parkinson’s Disease Questionnaire‑8.

p LEDD: levodopa equivalent daily dose.

### Barriers to Implementation and Practical Implications for Chronic DBS Care

Two patients were excluded from this study because of insufficient caregiver support and lack of digital literacy. Caregiver support is essential for patients with PD at these stages. Patients with PD may experience difficulty using small screens on tablet computers or smartphones due to motor impairments and cognitive dysfunction. These limitations may partly explain the relatively low agreement rates in the “Reliability” and “Interface quality” subdomains of the TUQ. These subdomains assess, “how easily the user can recover from an error and how the system provides guidance to the user in the event of an error” and “how pleasant the telemedicine technology or computer system was to use for the consumer,” respectively (Tables3  4) [24]. It remains difficult for clinicians to establish online consultation systems in hospitals because of limited medical resources. Administrative challenges include difficulties obtaining official prescriptions and a lack of medical insurance coverage [17]. The OUQP showed that it was sometimes difficult to provide rapid treatment resolution (31.3%; Table 5). This was, in part, due to the inability to touch the patients directly (eg, the inability to check the rigidity of the extremities). These challenges should be addressed to improve feasibility. Despite these concerns, it is surprising to see many patients satisfied with online sessions and inclined to use in the future as shown in the TUQ subdomain “Satisfaction and future use” (Table 4).

### Limitations

This study has some limitations. First, this was a single-arm longitudinal study without a comparator group, which limits causal inference and the ability to contextualize the observed changes. Long-term patients with PD equipped with DBS devices with no experience of online sessions are rare, making recruitment challenging; therefore, a single-arm design was the most feasible approach for this population. Second, the small sample size may have reduced the statistical power. Although it was determined a priori using conservative assumptions, the study may still have been underpowered to detect smaller effects. Third, the observation period for the online sessions was short and longer online sessions may yield different effects. Fourth, the study population was necessarily selective, as patients without caregiver support or with low digital literacy were unable to participate. This introduces a potential selection bias and limits the generalizability of our findings to more vulnerable groups. At the same time, patients who can realistically engage in remote DBS care in routine clinical practice are typically those with caregiver support and sufficient digital literacy; therefore, the present cohort also reflects the subset of patients who had undergone chronic DBS for whom online sessions are currently feasible. Finally, the findings apply specifically to patients with bilateral STN-DBS using the Infinity/NeuroSphere remote care system within a Japanese health care setting with adequate infrastructure, which further limits the applicability of our findings to other devices, stimulation targets, or health care systems. The absence of randomization, blinding, or parallel controls also raises the possibility of observer, performance, detection, and expectation bias. Thus, we must evaluate our results carefully because of these limitations.

### Conclusions

Sharma et al [27] described ideal candidates for online sessions as those who have a supportive caregiver, do not have cognitive or psychiatric impairment, or have impediments to in-person visits. Given that establishing a relationship with the DBS care team is essential for patients who had undergone de novo DBS [27] and that the postoperative levodopa equivalent daily dose reduction rate may be lower in patients managed online [15], online sessions might be most suitable for patients with chronically implanted DBS devices. The absence of statistically or clinically meaningful worsening in all aspects—with only a modest and clinically limited change in sleep status—in patients with PD and chronic DBS implantations supports the feasibility of incorporating online sessions, or a hybrid of in-clinic and online sessions, into DBS care. Such approaches could be considered as a potential means to alleviate the burden on patients and caregivers. Future studies incorporating validated sleep-specific instruments, such as the Pittsburgh Sleep Quality Index, objective measures including actigraphy, and longer follow-up periods, will be essential to determine whether the sleep changes identified here represent transient fluctuations or clinically relevant deterioration. Despite some limitations, the online sessions showed potential feasibility and an acceptable safety profile in this preliminary evaluation, suggesting their potential role in reducing the time and cost burden for patients.

## Supplementary material

10.2196/80223 Multimedia Appendix 1 Japanese version of Patient’s Global Impression of Change, Clinical Global Impression of Change, Telehealth Usability Questionnaire, and Online Usability Questionnaire for Programming.

10.2196/80223 Multimedia Appendix 2 Factors associated with changes in Multidimensional Evaluation Scale for Patient Impression Change—Japanese version item 3 (Sleep).

## Abbreviations

CGI-C: Clinical Global Impression of Change
DBS: deep brain stimulation
MDS-UPDRS-III: Movement Disorder Society Unified Parkinson's Disease Rating Scale Part III
MIC: minimally important change
MPIC-J: Multidimensional Evaluation Scale for Patient Impression Change—Japanese version
OUQP: Online Usability Questionnaire for Programming
PD: Parkinson disease
PGIC: Patient’s Global Impression of Change
ROC: receiver operating characteristic
STN: subthalamic nucleus
TUQ: Telehealth Usability Questionnaire
