# Supplementary material for: Influence of Online Sessions via a Deep Brain Stimulation Device: Prospective, Single-Arm, Longitudinal, Nonrandomized Self-Controlled Cohort Study
Source: JMIR Mhealth Uhealth. 2026 Jun 9;14:e80223. doi: 10.2196/80223 (PMC13249593; doi:10.2196/80223)
Supplement: Multimedia Appendix 1 [file mhealth-v14-e80223-s001.docx]

**Multimedia Appendix 1**

**Patient Global Impression of Change (PGIC) scale**

PGIC-1

ここ数か月（対面またはオンライン治療を開始して以来）、痛みや運動機能に伴う状態に関連する活動制限、症状、感情、および全体的な生活の質の変化はどれに該当しますか（1つだけ選択します）(Since beginning treatment at this clinic, how would you describe the change (if any) in ACTIVITY LIMITATIONS, SYMPTOMS, EMOTIONS and OVERALL QUALITY OF LIFE, related to your condition?)

1. 変化なし（または状態が悪化した）(No change or condition has got worse)
2. ほぼ同じ、ほとんど変化なし (Almost the same, hardly any change at all)
3. 少し良くなりましたが、目立った変化はありません (A little better, but no noticeable change)
4. いくらかは改善されましたが、変更による実際の違いはありません (Somewhat better, but the change has not made any real difference)
5. やや良くなり、わずかではあるが顕著な変化 (Moderately better, and a slight but noticeable change)
6. より良い、そして真の価値のある違いをもたらした明確な改善 (Better, and a definite improvement that has made a real and worthwhile difference)
7. 大幅に改善され、すべての違いをもたらしたかなりの改善 (A great deal better, and a considerable improvement that has made all the difference)

PGIC-2

同様に、当病院でのここ数か月（対面またはオンライン治療を開始して以来）の変化の度合いに合わせて以下の数字に〇をつけてください (In a similar way, please circle the number below, that matches your degree of change since beginning care at this clinic)

良くなった 変化なし 悪くなった (Much better) (No change) (Much worse)

0　　1　　2　　3　　4　　5　　6　　7　　8　　9　　10

Reference: Hurst H and Bolton J. Assessing the clinical significance of change scores recorded on subjective outcome measures. J Manipulative Physiol Ther 27:26-35, 2004. doi:10.1016/j.jmpt.2003.11.003

**Clinical Global Impression of Change (CGI-C)**

最終時点でのベースラインからの変化を下記の7段階で評価する。

ひとつだけ、当てはまるものを選んでください。

(Compared to the patient’s condition at the baseline, this patient’s condition is)

1. 著名悪化 (very much worse)
2. 中等度悪化 (much worse)
3. 軽度悪化 (minimally worse)
4. 不変 (no change from baseline)
5. 軽度改善 (minimally improved)
6. 中等度改善 (much improved)
7. 著名改善 (very much improved)

Reference: Guy W. *ECDEU Assessment Manual for Psychopharmacology, Revised*. Rockvill, MD: U.S. Dept. of Health, Education, and Welfare, Public Health Service, Alcohol, Drug Abuse, and Mental Health Administration, National Institute of Mental Health, Psychopharmacology Research Branch; 1976.

**Telehealth Usability Questionnaire (TUQ)**

- 1. かなり悪くなった　②悪くなった　➂少し悪くなった　④かわらなかった　⑤少しよくなった　⑥よくなった　⑦かなりよくなった。それぞれの質問でひとつ回答を選んで〇をつけてください。(Please select one response per item by marking a circle (○) around the option that best reflected their experience. The response scale consisted of seven categories: (1) markedly worsened, (2) worsened, (3) slightly worsened, (4) no change, (5) slightly improved, (6) improved, and (7) markedly improved.)

1)　遠隔医療システムは、医療サービスへのアクセスを改善してくれる。(Telehealth improves my access to healthcare services.)

いいえ(Disagree)←①　②　③　④　➄　⑥　⑦→はい(Agree)

2) 遠隔医療システムを使用することで、病院や専門医クリニックに行く時間を短縮できる。(Telehealth saves me time traveling to a hospital or specialist clinic.)

いいえ(Disagree)←①　②　③　④　➄　⑥　⑦→はい(Agree)

3) 遠隔医療システムは医療ニーズに応えてくれる。(Telehealth provides for my healthcare need.)

いいえ(Disagree)←①　②　③　④　➄　⑥　⑦→はい(Agree)

4) 遠隔医療システムは簡単に使用することができた。(It was simple to use this system.)

いいえ(Disagree)←①　②　③　④　➄　⑥　⑦→はい(Agree)

5)　遠隔医療システムの使い方は簡単に学ぶことができた。(It was easy to learn to use the system.)

いいえ(Disagree)←①　②　③　④　➄　⑥　⑦→はい(Agree)

6)　遠隔医療システムを使用すると、生産性が短期間で高まると思う。(I believe I could become productive quickly using this system)

いいえ(Disagree)←①　②　③　④　➄　⑥　⑦→はい(Agree)

7) 遠隔医療システムでのやり取りは楽しい。(The way I interact with this system is pleasant.)

いいえ(Disagree)←①　②　③　④　➄　⑥　⑦→はい(Agree)

8)　遠隔医療システムを使うことが好きである。(I like using the system.)

いいえ(Disagree)←①　②　③　④　➄　⑥　⑦→はい(Agree)

9)　遠隔医療システムはシンプルで理解しやすい。(The system is simple and easy to understand.)

いいえ(Disagree)←①　②　③　④　➄　⑥　⑦→はい(Agree)

10) 遠隔医療システムでは、期待通りのことをすべて行うことが可能である。(This system is able to do everything I would want it to be able to do.)

いいえ(Disagree)←①　②　③　④　➄　⑥　⑦→はい(Agree)

意思疎通の質について

11)　遠隔医療システムを使用することで、簡単に医師と話すことができた。(I can easily talk to the clinician using the telehealth system.)

いいえ(Disagree)←①　②　③　④　➄　⑥　⑦→はい(Agree)

12)　遠隔医療システムを使用することで、医師の声をはっきりと聞くことができた。(I can hear the clinician clearly using the telehealth system.)

いいえ(Disagree)←①　②　③　④　➄　⑥　⑦→はい(Agree)

13)　遠隔医療システムで自分自身を効果的に表現できたと思う。(I felt I was able to express myself effectively.)

いいえ(Disagree)←①　②　③　④　➄　⑥　⑦→はい(Agree)

14) 遠隔医療システムを使用することで、実際に会っているかのように医師の診療を受けることができた。(Using the telehealth system, I can see the clinician as well as if we met in person.)

いいえ(Disagree)←①　②　③　④　➄　⑥　⑦→はい(Agree)

15)　 遠隔医療システムによる診療は、対面診療と変わらないと思う。(I think the visits provided over the telehealth system are the same as in-person visits.)

いいえ(Disagree)←①　②　③　④　➄　⑥　⑦→はい(Agree)

16)　遠隔医療システムの使用中に間違えた場合でも、簡単かつ迅速に正常な状態に戻すことができた。(Whenever I made a mistake using the system, I could recover easily and quickly.)

いいえ(Disagree)←①　②　③　④　➄　⑥　⑦→はい(Agree)

17) 遠隔医療システムは、エラーメッセージを表示させて、問題解決法を分かりやすく教えてくれた。(The system gave error messages that clearly told me how to fix problems.)

いいえ(Disagree)←①　②　③　④　➄　⑥　⑦→はい(Agree)

18) 遠隔医療システムを使用した医師とのコミュニケーションに満足している。(I feel comfortable communicating with the clinician using the telehealth system.)

いいえ(Disagree)←①　②　③　④　➄　⑥　⑦→はい(Agree)

19)　遠隔医療は、医療サービスを受ける方法として許容できる。(Telehealth is an acceptable way to receive healthcare services.)

いいえ(Disagree)←①　②　③　④　➄　⑥　⑦→はい(Agree)

20)　今後もまた遠隔医療サービスを利用するつもりだ。(I would use telehealth services again.)

いいえ(Disagree)←①　②　③　④　➄　⑥　⑦→はい(Agree)

21) 遠隔医療システムのエラーメッセージは、問題を的確に修正するものである。(Overall, I am satisfied with this telehealth system.)

いいえ(Disagree)←①　②　③　④　➄　⑥　⑦→はい(Agree)

References: Parmanto B, Lewis AN, Graham KM, Bertolet MH. Development of the Telehealth Usability Questionnaire (TUQ). *Int J Telerehabilitation*. 8(1):3-10, 2016. doi:10.5195/ijt.2016.6196

**Online Usability Questionnaire for Programming (OUQP)**

オンライン有効性スコア

**医師による評価　〇をつけてください。(For physician-rated assessments, please mark a circle (○) around the most appropriate response option.)**

- - 1. **オーディオ・ビデオの接続ができたか (Ability to establish an audio-visual connection)**

いいえ(No)　はい (Yes)　どちらとも言えない (Neither)（理由 reason：　　　　　　　　　　　　）

**2) 意図した（プログラミングの）設定が行えたか (Achieving the clinical goals of a programming session)**

いいえ(No)　はい (Yes)　どちらとも言えない (Neither)（理由 reason：　　　　　　　　　　　　）

**3) オンライン診療のセッションを終了できたか (Ability to complete a session)**

いいえ(No)　はい (Yes)　どちらとも言えない (Neither)（理由 reason：　　　　　　　　　　　　）

**4) オンライン診療後に対面診療の必要性は減ったか (Reduction in the need for an in-person follow-up after a remote session)**

いいえ(No)　はい (Yes)　どちらとも言えない (Neither)（理由 reason：　　　　　　　　　　　　）

**5)　オンライン診療により迅速に治療上の問題が解決できたか (Providing rapid treatment resolution)**

いいえ(No)　はい (Yes)　どちらとも言えない (Neither)（理由 reason：　　　　　　　　　　　　）

**患者による評価 (For patient-reported assessments, please mark a circle (○) around the option that best represented their perception.)**

**6)　 オンライン診療に満足しているか (Satisfied with a remote session)**

いいえ(No)　はい (Yes)　どちらとも言えない (Neither)（理由 reason：　　　　　　　　　　　　）

**7) オンライン診療と対面診療のどちらを好むか (Preference for a remote session)**

いいえ(No)　はい (Yes)　どちらとも言えない (Neither)（理由 reason：　　　　　　　　　　　　）

**8)　 オンライン診療により迅速に治療上の問題を解決できたか (Providing rapid treatment resolution)**

いいえ(No)　はい (Yes)　どちらとも言えない (Neither)（理由 reason：　　　　　　　　　　　　）

**9)　　通院の負担低減　御回答ください。(Reduction in the burden of clinic visits)**

対面診療で要する通院時間、通院費（　　時間の短縮、通院費　約　　　　　　円の節約）(Time and cost required for in-person visits: e.g., reduction of approximately___minutes in travel time and saving of about___Yen in transportation costs )

その他の負担（介助者の負担、欠勤を要するなどの低減があれば下にご記入ください）(Other burdens: e.g., reduced burden on caregivers, fewer work absences, etc. Please describe below if applicable)

Reference: Deer TR, Esposito MF, Cornidez EG, Okaro U, Fahey ME, Chapman KB. Teleprogramming service provides safe and remote stimulation options for patients with DRG-S and SCS implants. *J Pain Res* 14:3259-3265, 2021. doi:10.2147/JPR.S332966
