# Supplementary material for: Influence of Online Sessions via a Deep Brain Stimulation Device: Prospective, Single-Arm, Longitudinal, Nonrandomized Self-Controlled Cohort Study
Source: JMIR Mhealth Uhealth. 2026 Jun 9;14:e80223. doi: 10.2196/80223 (PMC13249593; doi:10.2196/80223)
Supplement: Multimedia Appendix 2 [file mhealth-v14-e80223-s002.docx]

Multimedia Appendix 2

Table S1. Correlations between the change in MPIC item 3 and baseline factors.

| Baseline factors | *ρ* | *P*-value |
| --- | --- | --- |
| Sex* | -0.074 | 0.79 |
| Age | -0.28 | 0.29 |
| Hoehn-Yahr stage | 0.016 | 0.95 |
| MDS-UPDRS motor score | -0.20 | 0.45 |
| Disease duration | 0.23 | 0.40 |
| Duration after surgery | -0.080 | 0.77 |
| Distance from the residence | 0.26 | 0.32 |
| Travel time to the clinic | -0.23 | 0.39 |
| travel expenses for medical visits | 0.16 | 0.55 |
| Number of caregivers | 0.063 | 0.82 |
| MPIC-J item1 pre-online | 0.14 | 0.59 |
| MPIC-J item2 pre-online | 0.11 | 0.68 |
| MPIC-J item4 pre-online | 0.33 | 0.21 |
| MPIC-J item5 pre-online | 0.17 | 0.52 |
| MPIC-J item6 pre-online | 0.26 | 0.34 |
| MPIC-J item7 pre-online | -0.028 | 0.92 |
| MPIC-J item8 pre-online | -0.051 | 0.85 |

*Point‑biserial correlation coefficients (*r*) were used. For all other variables, Spearman’s rank correlation coefficients (*ρ*) were calculated.

Table S2. Correlations between the change in MPIC item 3 and exposure‑related factors.

| Exposure-related factors | *ρ* | *P*-value |
| --- | --- | --- |
| Duration of in-person sessions | 0.0077 | 0.98 |
| Duration of online sessions | 0.71 | 0.0021* |

Table S3. Correlations between the change in MPIC item 3 and change-based variables.

| Change-based variables (*Δ*) | *ρ* | *P*-value |
| --- | --- | --- |
| *Δ*MDS-UPDRS-3 of in-person sessions | 0.52 | 0.038 |
| *Δ*MDS-UPDRS-3 of online sessions | -0.40 | 0.12 |
| *Δ*MPIC-J item1 | 0.24 | 0.36 |
| *Δ*MPIC-J item2 | 0.44 | 0.086 |
| *Δ*MPIC-J item4 | -0.20 | 0.46 |
| *Δ*MPIC-J item5 | 0.0025 | 0.99 |
| *Δ*MPIC-J item6 | -0.14 | 0.61 |
| *Δ*MPIC-J item7 | 0.54 | 0.031 |
| *Δ*MPIC-J item8 | 0.19 | 0.48 |
